# Host Defence Peptides in Diabetes Mellitus Type 2 Patients with Periodontal Disease. A Systematic Review

**DOI:** 10.3390/diagnostics11122210

**Published:** 2021-11-26

**Authors:** Muhammad Saad Shaikh, Muhammad Sohail Zafar, Farhan Saleem, Ahmad Alnazzawi, Mohid Abrar Lone, Syed Jawad Ali Bukhari, Zohaib Khurshid

**Affiliations:** 1 Department of Oral Biology, Sindh Institute of Oral Health Sciences, Jinnah Sindh Medical University, Karachi 75510, Pakistan; drsaadtanvir@gmail.com; 2 Department of Restorative Dentistry, College of Dentistry, Taibah University, Al Madinah Al Munawwarah 41311, Saudi Arabia; 3 Department of Dental Materials, Islamic International Dental College, Riphah International University, Islamabad 44000, Pakistan; 4 General Dentistry, Edith Cowan University, Perth, WA 6027, Australia; dr.farhansaleem13@gmail.com; 5 Department of Substitutive Dental Sciences, College of Dentistry, Taibah University, Al Madinah Al Munawwarah 41311, Saudi Arabia; alnazzawi@gmail.com; 6 Department of Oral Pathology, Sindh Institute of Oral Health Sciences, Jinnah Sindh Medical University, Karachi 75510, Pakistan; mohid.lone@jsmu.edu.pk; 7 Centre for Addiction and Mental Health, University of Toronto, Toronto, ON M5S 1A1, Canada; jawadali83@hotmail.com; 8 Department of Prosthodontics and Dental Implantology, College of Dentistry, King Faisal University, Al-Ahsa 31982, Saudi Arabia; zsultan@kfu.edu.sa

**Keywords:** periodontal disease, diabetes mellitus, antimicrobial peptides, risk factor

## Abstract

The aim of the study was to critically assess and review the latest evidence relating the associations between host defence peptides (HDPs), periodontal diseases (PD) and diabetes mellitus type 2 (DM2). To explore studies on HDPs, periodontal disease, and DM2, researchers utilised specific key phrases to search the electronic databases PubMed (National Library of Medicine), Embase (Ovid), Medline (EBSCO), and Dentistry and Oral Sciences (EBSCO). Quality assessment was conducted by means of the Newcastle Ottawa scale and the Systematic Review Centre for Laboratory Animal Experimentation (SYRCLE) tool. Following a thorough screening process, a total of 12 papers (4 case-control, 6 cross-sectional, 1 animal, and 1 in vitro) fulfilled the selection criteria and were included. The majority of research found that HDPs were upregulated in DM2 patients with PD. Three investigations, however, found that HDPs were downregulated in DM2 patients with PD. HDPs play a part in the pathophysiology of PD and DM2. Nonetheless, more human, animal and laboratory investigations are needed to fully understand validation of the link, as the evidence is limited. Understanding HDPs as common moderators is critical, aimed at unlocking their potential as therapeutic and diagnostic agents.

## 1. Introduction

Dental plaque biofilm is the primary cause of periodontal disease (PD), leading to progressive deterioration of periodontal apparatus [1]. PDs are caused by a series of events involving the non-specific defence mechanism (innate) and antigen-specific immune response (acquired) [2]. Host defence peptides (HDPs) that are largely produced by epithelial cells and the acute inflammatory cells, i.e., neutrophils, and have antimicrobial characteristics are a vital constituent of the non-specific defence system. [3,4]. Human saliva as well as gingival crevicular fluid (GCF) contain plentiful HDPs, comprising human alpha- (hADs) and beta-defensins (hBDs), cathelicidin (hCAP18/LL-37), adrenomedullin, histatin, and others [5,6,7,8]. These HDPs play a significant role in the maintenance of the periodontal pocket and help in combating microbes.

Aside from their well-known antibacterial activity, they also contribute to innate and adaptive immunity by boosting phagocytosis, decreasing proinflammatory cytokine production, and modulating the complement system [9]. They operate as immune cell chemoattractants and promote wound healing and angiogenesis [10]. Despite their well-defined impacts on immunity, it is unclear how periodontitis and associated variables influence antimicrobial peptide levels [11,12,13,14].

Diabetes mellitus type 2 (DM2) is a chronic metabolic condition characterised by insulin resistance. The disease occurrence is anticipated to be above 400 million people (20–79 years old) globally, with 629 million expected by 2045 [15]. It is also regarded as a modifiable periodontitis risk factor [16,17]. The immune-inflammatory axis is disrupted in both PD and DM2. To untangle their interaction and develop treatment strategies, a comprehensive knowledge of the fundamental biological processes is required.

HDPs have been identified as important players in immune modulation in this regard [18,19]. Studies have shown that HDPs are involved both in the initiation/progression of PD and in the immune-inflammatory reactions that characterise PD risk factors such as DM2 [20,21] and other risk factors such as smoking [22], psychological stress [23], and human immunodeficiency virus (HIV) [24]. However, it is yet unknown how and if HDPs have a role in the relationship between PD and DM2. This systematic review was aimed at giving the recent available information on the HDPs’ involvement in PD and one of its major risk factors, DM2.

## 2. Materials and Methods

### 2.1. Focused Question

“Is there any likely association of HDPs in patients with PD and DM2?”

This systematic review was devised in accordance with the most recent Preferred Reporting Items for Systematic reviews and Meta-Analyses (PRISMA) recommendations [25].

### 2.2. Literature Search

A total of four electronic databases, PubMed (National Library of Medicine, Maryland, United States of America), Embase (Ovid), Medline (EBSCO), and Dentistry and Oral Sciences (EBSCO) were used to conduct literature searches from January 1990 to January 2021. The following search method was used to find relevant literature on the association between HDPs, PD, and DM, using Boolean operators; “Periodontal disease” OR “Chronic periodontitis” OR “periodontitis” AND “diabetes” OR “diabetes mellitus” OR “diabetes mellitus type 2” AND “antimicrobial peptide” OR “defensin” OR “cathelicidin”. Further literature was found by searching the bibliographical lists of pertinent articles. Relevant papers were found and rigorously analysed in order to interpret the findings. Table S1 represents search tracking of this review paper.

### 2.3. Screening and Selection Process

The screening process was performed by two different authors (M.S.Z. and M.S.S.). Screening was conducted in three phases, i.e., titles, abstracts and full texts. The Cohen Kappa score *(κ)* [26] was used to assess the authors’ inter-rater agreement. In the event of a disagreement or discrepancy, a third reviewer was consulted to reach a consensus.

**Figure 1:**
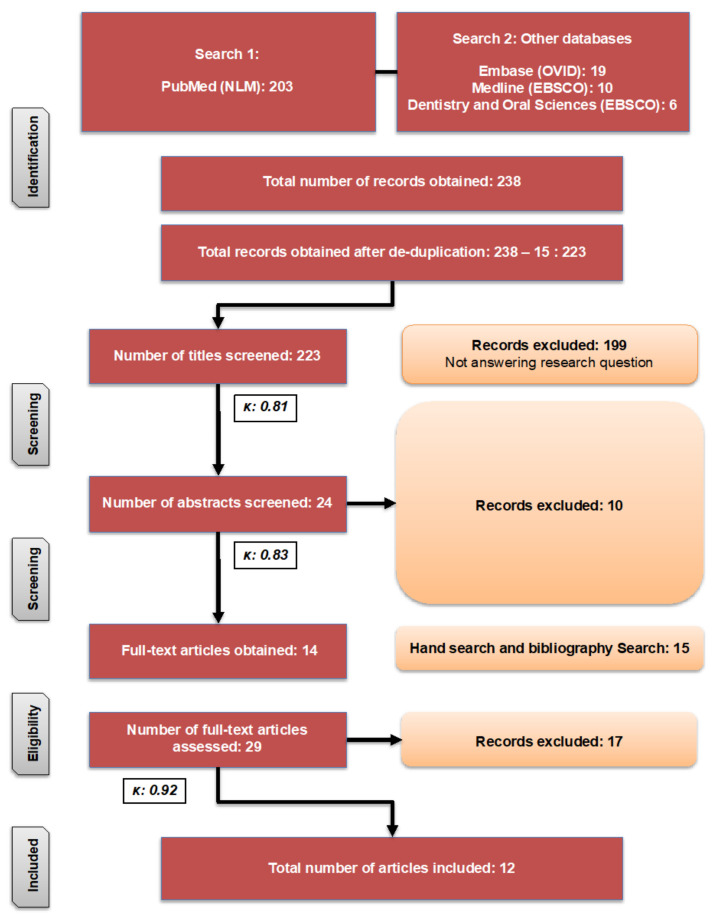
Search strategy used to search articles.

Excluded were in vivo studies on HDPs’ expression in GCF/saliva/gingival tissue (GT) of DM2 and periodontally healthy individuals. Furthermore, in vivo studies on HDPs’ expression in GCF/saliva/GT of patients with periodontitis but not having DM2 were excluded. However, studies on HDPs’ expression in GCF/saliva/GT of individuals with periodontitis suffering from DM2 were included. Laboratory investigations on the effect of DM on expression of HDP in human oral epithelial/keratinocyte cells were also included. Figure 1 depicts the search process.

### 2.4. Eligibility Criteria and Data Extraction

The search was restricted to papers written in English. In vivo studies involving humans or animals of all ages, regardless of gender, were included. Also included were in vitro research on cultured cells, human gingival keratinocytes or epithelial cells. Languages other than English, review articles, studies presented as abstracts of scientific conferences, and other unpublished papers were all excluded. The data extraction comprised the name of author, study year, the tissue/fluid analysed in the study, the investigated HDP, details regarding participants, main results of the study, and the change model.

### 2.5. Quality Appraisal

The quality appraisal was implemented by means of the Newcastle Ottawa scale (NOS) for case-control papers [27], NOS modified for cross-sectional research [28], and the Systematic Review Centre for Laboratory animal Experimentation (SYRCLE) tool for animal studies [29]. For case-control research, NOS comprises eight items with three domains, with a maximum score of nine. Because a standard measure for what comprises a high-quality study has not been generally recognised yet, a score of seven or greater was rendered to be a high-quality study.

For the NOS modified for cross-sectional studies, a score of 9–10 was judged to be a very good study, a score of 7–8 was classified as a good study, a score of 5–6 was classified as a satisfactory study, and score of 0–4 was judged to be an unsatisfactory study. In the case of the SYRCLE tool, six distinct domains were evaluated in a study. A paper was classified as having low bias risk if each domain had a low bias risk, an unclear bias risk if any one domain had an unclear bias risk, and a high bias risk if any one domain had a high bias risk. Two reviewers (M.S.Z. and M.S.S.) independently carried out the data extraction and quality assessment process.

## 3. Results

### 3.1. Search Results

A comprehensive search yielded 223 articles (excluding duplicates). Following the review of titles, abstracts, and full-texts, 14 papers were available. Furthermore, a manual search resulted in the inclusion of 15 additional articles. After excluding 17 of the 29 articles, 12 papers were chosen for qualitative analysis (Figure 1). Table 1 displays the study’s characteristics. The meta-analysis was not possible as a result of heterogeneity of the results of the papers included. The Cohen’s Kappa score (***κ***) was found to be 0.81 (titles screening), 0.83 (abstract screening) and 0.92 (full-text screening); that was based on the usually cited scale for Kappa score interpretation that advocates strong to almost perfect reviewers’ inter-rater agreement [26].

From a total of 12 studies, 10 were observational studies (6 cross-sectional [30,31,32,33,34,35] and four case-control [36,37,38,39]), 1 was an animal study [40], and 1 was an in vitro study [41]. Different fluids/tissues were used for the HDPs’ analysis, including GCF [30,31,34,36,37,38], saliva [33,35,39], GT [32], and human oral epithelial cells [41]. However, one study [40] used both blood and GT for the analysis. Three studies investigated adrenomedullin [30,36,38], two studies investigated HBD 1 [33,34], and study each investigated hBD 1 and hBD 3 [31], hBD 2, hBD 3 and hCAP18/LL-37 [32], hBD 2 [35], hCAP18/LL-37 [37], hBD 1, hBD 2, hBD 3 and hCAP18/LL-37 [39], and hBD 3 [40] and lipocalin 2 [41].

For the human-based studies, the total number of patients was 1011. However, for the one animal study [40], total of eight monkeys were utilized. From the 12 studies, 9 showed greater levels of HDP in the DM2 group with PD [30,31,32,33,35,36,38,40,41]. In one study, the healthy control group showed more increased levels of HDP than the DM2 group with PD [34,37]. Interestingly, one study showed greater levels of hCAP18/LL-37 and decreased hBD 1, hBD 2, and hBD 3 levels in DM2 with the PD group than the CP group [39].

### 3.2. Quality Appraisal

**Figure 2:**
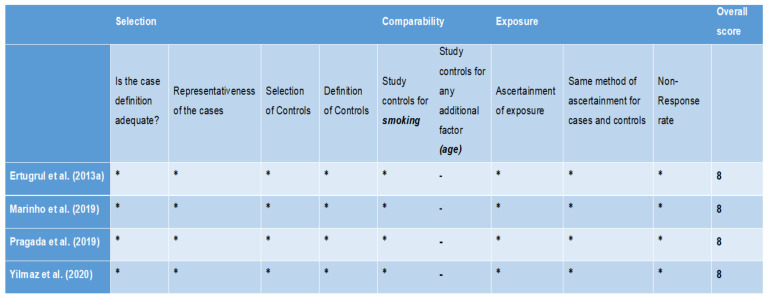
Quality appraisal of case-control studies via Newcastle Ottawa scale. A study can be awarded a maximum of one star (*) for each numbered item within the Selection and Exposure categories. A maximum of two stars can be given for Comparability.

**Figure 3:**
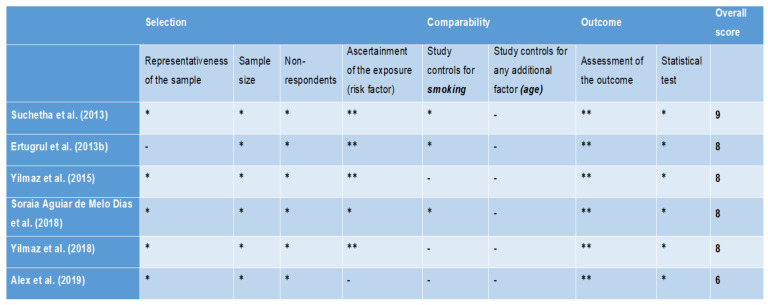
Quality appraisal of cross-sectional studies via modified Newcastle Ottawa scale. A study can be awarded a maximum of one star (*) for each numbered item within the Comparability categories. A maximum of five stars can be given for the Selection category (two stars for the Ascertainment of the exposure) and three stars can be given for the Outcome category (two stars for the Assessment of outcome).

**Figure 4:**
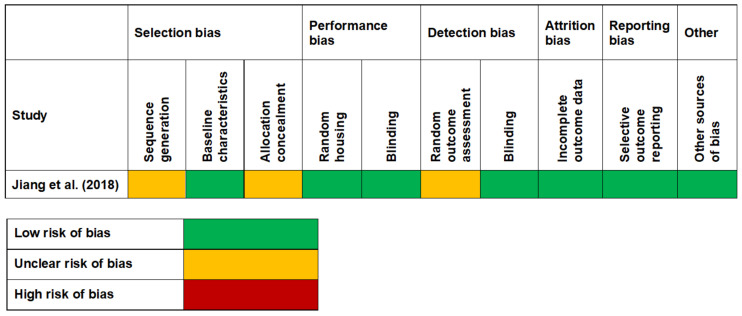
Quality appraisal of animal study via SYRCLE tool.

All the studies [36,37,38,39] evaluated by NOS for cross-sectional studies scored more than 7, suggesting high quality of the studies (Figure 2). For studies assessed by the NOS modified for cross-sectional studies, 1 study [30] scored 9 points (very good study), 4 studies [31,32,33,34] scored 8 points (good studies), and 1 study [35] scored 6 points (satisfactory study) (Figure 3). The only animal study [40] was categorised to have unclear bias risk, as assessed by the SYRCLE tool (Figure 4).

## 4. Discussion

Diabetes mellitus is a group of metabolic disorders characterised by chronic hyperglycaemia, causing dysfunction in carbohydrate, protein and fat metabolism due to a complete lack of insulin or its effect [42]. Diabetes Mellitus type 1 (DM1) is insulin-dependent and is supposed to be an autoimmune disorder. On the other hand, DM2 is not insulin-dependent and has chronic inflammatory process as a main component of its pathophysiology [43]. Because chronic periodontitis (CP) is also a chronic inflammation, the link between PD and DM2 is much greater than the link between PD and type 1 DM [31]. It is widely acknowledged that there is a bi-directional relationship between PD and DM2, and that DM2 frequently aggravates the devastating process of PD, and vice versa [44].

Several studies explored the biological correlation between DM2 and PD. In certain investigations, DM2 has been linked to a decrease in tissue HDP synthesis [45,46], although one study reported an insignificant difference in serum HDP levels [47]. PD and DM2 both are complicated disorders comprising a genetical constituent. Decreased hBDs and greater hADs levels have been observed in DM2, related to genetics; correspondingly, lower hBD 2 has been observed in severe CP in regard to genetical copy number diversity [48,49]. Furthermore, the function of defensins in mediating tissue destruction in DM2 complications has been discussed [48]. These findings potentially point to shared genetic susceptibility factors underpinning poor regulation and function of HDP in DM2 as well as PD. Aside from genetical susceptibility, hyperglycaemia and PD may alter HDP expression as well as function in epithelial cells independently [45]. The chemicals dicarbonyls methylglyoxal and glyoxal are generated in response to hyperglycaemia and have been shown to degrade hBD 2; the suppression in immunity emphasises the enhanced sensitivity to cellular invasion by Gram-negative bacteria observed in DM2 [50].

Several human investigations were conducted to determine the involvement of HDPs in people with PD and DM2 (Table 1). Despite the fact that this research involved distinctive types of samples (GCF and GTs) and targeted several different HDPs, they all yielded comparable results, suggesting upregulation of HDP in DM2 with periodontitis, with the exception of three studies that revealed downregulation of hCAP18/LL-37 [37], hBD 1 [34] and hBD 1, hBD 2, and hBD 3 [39]. The hBD 1 levels in GCF were considerably greater in patients with DM2 and CP than in systemically healthy patients with CP alone [31]. In contrast, a comparable study that looked at hBD 2 levels in GT [32] did not find significant differences between the two groups. The hBD 3 expression in GCF [31] and GT [32,40] have revealed similar outcomes, signifying that DM2 may upregulate hBD 3 levels in individuals with PD. Likewise, hCAP18/LL-37 [32,39], lipocalin 2 [41], and adrenomedullin [30,36,38], a multifunctional vasopeptide, have been overexpressed in individuals with DM2 with PD. However, a paucity of experimental substantiation of the DM effects on the molecular mechanisms behind expression of HDP in GT still exist.

The animal study [40] investigating the expression of hBD 3 in GT showed upregulation in DM2 rhesus monkeys compared to the control group. The result of this animal study was consistent with research by Yilmaz et al. (2015) [32] that was conducted on humans and also demonstrated overexpression of hBD 3 in GT.

Likewise, an in vitro study [41] showed that the expression of lipocalin 2 in human oral epithelial cells was increased due to advanced glycation end products (AGEs), which is a major factor that causes DM2 complications and induces inflammatory responses in some systemic tissues.

A potential explanation of the HDPs’ upregulation in DM with PD is the accumulation of AGEs forming due to chronically elevated blood levels of glucose, and enhanced AGE serum levels resulting in simultaneous secretion into the GCF [51]. Increased AGE levels not only cause thickening in periodontal tissue basal membrane and vascular injury, but they also harm endothelial cells, polymorphonuclear neutrophils, monocytes, and macrophages [52]. The resulting loss of vascular structure and cell dysfunction impedes chemotactic factor migration and activity, reducing leukocytes’ ability to protect periodontal tissues from the pathogens’ effect [31,36]. This results in an elevated periodontal pathogen load, leading to an augmented gingival HDP release to fight infection and halt the loss of periodontal tissue. Nevertheless, such putative protective functions of elevated HDPs remain insufficient to restrict inflammation of periodontal tissues and promote appropriate wound healing [31,32,36].

Three investigations, on the other hand, found HDP downregulation in DM2 with CP. This could be explained by the fact that hyperglycaemic circumstances inhibit expression of hBD 3 at the mRNA as well as protein levels in human keratinocytes [45]. Excess AGE production inhibited P38 mitogen-activated protein kinases’ (p38MAPK) signalling, which explained their findings. Another in vitro investigation found that while ageing can cause a transitory rise in p38MAPK signalling, at greater levels ageing causes HDPs to be less expressed [53]. Other research, however, has found elevated amounts of hBDs in DM2 [31,32,33,35,40]. These changes may be due to different sources of sampling such as gingiva, GCF, saliva, and participant DM2 management.

Although these few studies attempted to identify the possible relationships among HDPs, PD, and DM2, the fundamental variables and mechanisms remain unknown. Some questions about this matter are yet unanswered: How can DM2 and PD processes participate in HDP production disruption? What role do HDPs play in poor glycaemic control and consequent PD? Furthermore, the effects direction of HDP levels on severity of DM2 and PD remains unknown. More follow-up studies with a bigger sample size are necessary to verify HDPs as a biomarker in PD and DM2 advancement, as well as to explain the vagueness of its involvement. In addition, future research should include in vitro studies in human gingival epithelial cells/keratinocytes investigating the mechanism of DM2 on HDP expression. Furthermore, randomised controlled clinical trials of periodontal treatment should be conducted comparing DM2 and CP with a CP-only group and healthy patients to explore the consequence of periodontal therapy on HDP levels in these individuals.

Few limitations were encountered in this review. First, no meta-analysis could have been conducted as a result heterogenous data such as study designs, population assessed, tissue type analysed, and finally the HDP investigated in the study. Secondly, only one in vitro and one animal study were retrieved after thorough search, showing the scarcity of exploration of HDPs in DM2 and PD in these study designs. Thirdly, the one animal study included was classified to be of unclear risk on the SYRCLE tool. Fourthly, the quality appraisal of the one in vitro study could not be performed as there is no validated tool available. Lastly, a majority of studies included in this review were the cross-sectional and case-control design, and that did not allow us to monitor possible fluctuations in the secretions of HDPs in response to changes in the glycaemic status, which can be considered as a limitation.

## 5. Conclusions

A hyperglycaemic environment may result in periodontal tissue destruction by accelerating inflammatory response and weakening the defence system in periodontal tissue. According to the current literature, HDPs have a potential mechanistic linkage between PD and DM2. Those data, however, are relatively restricted, and more human, animal, and laboratory investigations are required to elucidate the authenticity of these connections. A better understanding of HDPs as a conjoint mediator is critical for determining their relevance for treatment or diagnostic possibilities, given the two-way association between PD and DM2.

## Figures and Tables

**Table 1:** Characteristics of studies demonstrating association between HDP, PD, and DM2.

Author/Year	Analysed Fluid/Tissue	Investigated HDP	Study Participants	Outcomes	HDP Levels (Increase or Decrease)
***Cross-sectional studies***					
Suchetha et al. (2013) [30]	GCF	Adrenomedullin	Patients with DM2 + CP: 45 Patients with CP with no DM2: 30 Periodontally and systemically healthy patients (control): 15	(1) DM2 + CP group > other groups (*p* < 0.001) (2) CP group > healthy controls (*p* < 0.001)	Increase
Ertugrul et al. (2013b) [31]	GCF	hBD 1 hBD 3	Patients with DM2 and gingivitis: 20 Patients with DM2 and CP: 20 HG patients: 20 HCP patients: 20	For both hBD 1 and 3: (1) DM2 and CP group > other groups (*p* < 0.05) (2) DM2 and gingivitis group > HG group (*p* < 0.05) (3) DM2 and gingivitis group > HCP group (*p* < 0.05) (4) HCP group > HG group (*p* <0.05)	Increase
Yilmaz et al. (2015) [32]	GT	hCAP18/LL-37 hBD 2 hBD 3	Patients with DM2 + GP: 14 Healthy patient with GP: 11 Healthy patients (control): 13	(1) DM2 + GP group > healthy controls (hCAP18/LL-37: *p* = 0.002; hBD 2: *p* = 0.005; hBD 3: *p* = 0.007); (2) Insignificant difference between GP group and DM2 + GP group (*p* > 0.05); (3) Insignificant difference between healthy controls and GP group (*p* > 0.05)	Increase
Soraia Aguiar de Melo Dias et al. (2018) [33]	Saliva	hBD 1	Patients with DM2 + CP: 116 Patients with CP with no DM2: 95 Periodontally and systemically healthy patients: 69	(1) DM2 + CP > healthy controls (*p* < 0.05) (2) CP > healthy controls (*p* < 0.05)	Increase
Yilmaz et al. (2018) [34]	GCF	hBD 1	Patients with DM2 + GP: 21 Systemically healthy patient with GP: 18 Periodontally healthy patients with DM2: 18 Periodontally and systemically healthy patients (control): 24	Healthy controls > other groups (*p* < 0.05)	Decrease
Alex et al. (2019) [35]	Saliva	hBD 2	Patients with DM2 + GP: 20 Systemically healthy patient with CP: 20	DM2 + GP > CP group (*p* < 0.05)	Increase
***Case-control studies***					
Ertugrul et al. (2013a) [36]	GCF	Adrenomedullin	Patients with DM2 + CP: 21 Healthy patients with DM2: 21 Patients with CP with no DM2: 21 Periodontally and systemically healthy patients (control): 21	(1) DM2 + CP group > CP group, DM2 group and healthy controls (*p* < 0.05) (2) DM2 group > CP group and healthy controls (*p* < 0.05) (3) CP group > healthy controls (*p* < 0.05)	Increase
Marinho et al. (2019) [37]	GCF	hCAP18/LL-37	Patients with DM2 + CP: 5 Periodontally health patients with DM2: 5 Patients with CP with no DM2: 5 Periodontally and systemically healthy patients (control): 5	(1) DM2 + CP < healthy controls (*p* < 0.05) (2) CP < healthy controls (*p* < 0.05)	Decrease
Pragada et al. (2019) [38]	GCF	Adrenomedullin	Patients with DM2 + CP: 30 Periodontally health patients with DM2: 30 Patients with CP with no DM2: 30 Periodontally and systemically healthy patients (control): 30	(1) DM2 + CP > other groups (*p* < 0.05) (2) DM2 group > healthy controls (*p* < 0.05) (3) CP group > healthy controls (*p* < 0.05)	Increase
Yilmaz et al. (2020) [39]	Saliva	hCAP18/LL-37 hBD 1 hBD 2 hBD 3	Patients with DM2 + CP: 63 Periodontally health patients with DM2: 58 Patients with CP with no DM2: 29 Periodontally and systemically healthy patients (control): 28	(1) DM2 + CP group > healthy controls (hCAP18/LL-37: *p* < 0.05) (2) CP group > other groups (hBD 1: *p* < 0.05; hBD 2: *p* < 0.05; hBD 3: *p* < 0.05)	Increase for hCAP18/LL-37 Decrease for hBD 1, 2 and 3
***Animal study***					
Jiang et al. (2018) [40]	Blood GT	hBD 3	Monkeys with DM2 + PD: 5 Periodontally and systematically healthy monkeys (control): 3	DM2 + PD > control group (*p* < 0.05) Serum blood level of hBD 3 and gingival expression of hBD 3 mRNA in DM monkeys were considerably higher than in the healthy controls.	Increase
***In vitro study***					
Kido et al. (2020) [41]	HOEC	Lipocalin 2	-	AGEs increased the expression levels of lipocalin 2 in HOEC	Increase

Advanced glycation end products (AGEs); Cathelicidin (hCAP18/LL-37); Chronic periodontitis (CP); Diabetes mellitus type 2 (DM2); Healthy patients with gingivitis (HG); Healthy patients with chronic periodontitis (HCP); Human β-defensin (hBD); Human oral epithelial cells (HOEC); Generalised periodontitis (GP); Gingival crevicular fluid (GCF); GT (Gingival tissue); Periodontal disease (PD).

## Data Availability

Data supporting the reported results can be found at PubMed (National Library of Medicine), Embase (Ovid), Medline (EBSCO), and Dentistry and Oral Sciences (EBSCO databases.

## Supplementary Materials

The following are available online at https://www.mdpi.com/article/10.3390/diagnostics11122210/s1, Table S1: Search tracking.
